# Reconstituting alternative life using the test-bed of cell-free systems

**DOI:** 10.1098/rstb.2024.0293

**Published:** 2025-10-02

**Authors:** Maud Hofmann, Farouk Abdo, Olivier Borkowski, Manish Kushwaha

**Affiliations:** ^1^ Université Paris-Saclay, INRAE, AgroParisTech, Micalis Institute, Jouy-en-Josas 78352, France

**Keywords:** cell-free systems, synthetic life, origin of life, prototyping

## Abstract

The current form of life on Earth is the outcome of a series of biochemical events that led to the formation of early life with a specific molecular composition encoding its many functions. However, other trajectories of events with widely different outcomes could have led to alternative forms of life with different compositions. Although these ‘roads not taken’ have been hypothesized in Origins of Life research for long, only recently have advances in several life technologies enabled us to experimentally explore them. Here, we discuss how one such technology, cell-free systems (CFS), which offers a promising avenue for reconstitution of specific biological functions in a controlled *in vitro* environment. Breaking free of the complex biochemical interactions of the closed cellular compartment facilitates testing of engineered alternatives of these functions, in whole or part, reconstituted using both natural and synthetic orthogonal components. In this perspective, we focus on how CFS has enabled characterization and reconstitution of several steps of biological information transfer using alternative machinery (xeno nucleic acids, RNA polymerases, ribosomes, non-canonical amino acids, transfer RNA synthetases, mirror-image biomolecules), including in alternative biochemistries and compartments, identifying the applicable constraints. Once these alternative functions have been characterized and co-optimized, how do we foresee their reintegration into alternative life forms of the future?

This article is part of the theme issue ‘Origins of life: the possible and the actual’.

## 1. Introduction

Life as we know it arose from a series of biochemical events leading to the last universal common ancestor (LUCA), with a unique molecular composition encoding its functions [1,2]. However, alternative trajectories—such as xeno nucleic acids (XNAs), different transcription-translation systems, or mirror-image biomolecules—could have led to entirely different life forms. These alternatives either failed or were outcompeted, resulting in all known life evolving from LUCA [3]. How might these ‘roads not taken’ have been different from current life? In order to answer this question, we must first define life’s core functions. Gánti’s chemoton model and other studies identify these as information replication, metabolism and compartmentalization [4,5]. While these functions may be abstracted as distinct or modular, in living cells, they are intricately tied to each other. Consequently, alternative versions of these functions must be mutually compatible in order to generate fully functional alternative life. Before the full array of alternative functions could be assembled together, each of them needs to be tested individually. Cell-free systems (CFS) retain aspects of cellular functions in non-living environments [6], making them ideal test-beds for alternative life functions, even if they do not lead to a self-replicating cell [7].

**Figure 1.**
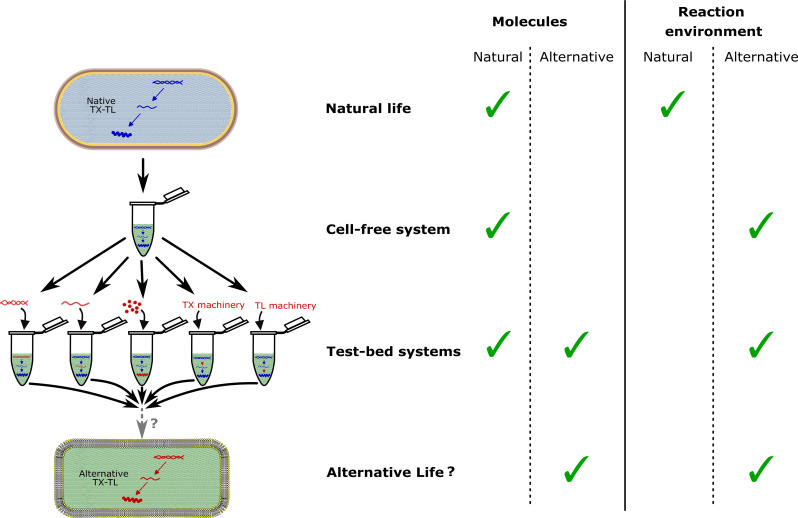
Illustration of the characteristics of natural organisms, cell-free systems, test-bed systems and potential alternative life. Left: the blue background represents the natural reaction environment, while the green background represents the alternative reaction environment used in cell-free systems. The blue shapes represent natural molecules (DNA, mRNA, and proteins), whereas the blue arrows indicate natural transcription and translation machineries. The red shapes and arrows represent alternative molecules and machineries, respectively. Right: the table outlines the types of molecules and reaction environments that constitute natural life, cell-free systems, test-bed systems and potential alternative life.

In this direction, synthetic biologists have made significant advances in building life-like systems using a bottom-up approach [8], constructing cells from non-living components [9–12]. This approach includes using purified cellular components in appropriate buffers or employing cellular lysates containing cytoplasmic elements (figure 1). Reconstructing cells from scratch allows for alternative pathways, potentially leading to synthetic organisms distinct from their original cellular sources [13,14]. Investigating these alternative pathways not only enhances our understanding of cellular construction and evolution but also enables the design of novel biological systems with unique properties and functions [8]. By exploring alternative ways to build a living cell, we can quantitatively assess their complexity, providing insights into their evolutionary potential and functional diversity [14,15].

### (a) Cell-free systems for bottom-up reconstruction of biological functions

CFS are versatile platforms that enable biological reactions to occur outside living cells [16]. They can be classified into protein synthesis using recombinant elements (PURE) systems and lysate-based systems. PURE systems are highly defined, consisting of purified enzymes and ribosomes for precise control, ensuring reproducibility and minimal interference [17–19]. Lysate-based systems, derived from crude cellular extracts, such as from *Escherichia coli* cells, retain essential enzymes, metabolites and molecular machinery for gene expression [20–22], and are highly efficient, scalable and cost-effective [6,21–24].

Despite their several advantages, CFS often lack key cellular functions like adaptation, homoeostasis and spatial organization owing to loss of critical components, such as proteins of the cell membrane and cellular RNAs, during lysate preparation [6]. Recent advances aim to restore these functions by incorporating synthetic components or engineered devices, to enable more complex synthetic biology applications [6,25,26]. Future research will seek to create life-like out-of-equilibrium systems capable of metabolism and information processing, similar to natural living organisms [27,28]. Such advancements may enable energy generation, compartmentalization and even evolution to occur outside of living organisms, mirroring natural biological functions without deriving from existing life forms.

CFS also offer a unique platform to investigate fundamental biological processes, including information transfer and sub-cellular organization, in a simplified and controllable setting. In natural cells, genetic information flows from DNA to RNA and then to proteins—a central dogma that drives all cellular functions [29–31]. CFS recapitulates these information transfer steps outside of living cells, enabling the precise manipulation of transcription and translation processes to better understand and optimize gene expression mechanisms [23,32–34]. It facilitates testing of new metabolic pathways as well as mixing and matching of metabolism from different organisms [34,35]. Moreover, CFS allows exploration of sub-cellular organization via protocells or organelle-like structures [36,37]. These compartments can mimic the spatial segregation of biological functions, facilitating complex biochemical reactions in localized environments [38–41].

By using these capabilities, CFS enable the exploration of combinations of spaces that are difficult or impossible to achieve in living cells [42–45]. For example, modified metabolism or synthetic pathways can be investigated in a highly modular and flexible manner [46,47]. Artificial compartments further enable the study of membrane-bound processes, energy generation and metabolic networks, similar to how organelles function in eukaryotic cells [48–50]. Importantly, just because specific functions and sub-functions are able to operate in isolation in CFS does not guarantee their composability into the larger synthetic cell owing to the many inherent constraints and incompatibilities associated with interfacing the modules. However, CFS provides a powerful platform to dissect and re-engineer the key features of life, pushing the boundaries of synthetic biology and the development of alternative life forms.

### (b) Design gap between synthetic protocells and natural cells

CFS plays a crucial role in narrowing the ‘SynCell design gap’ by providing a versatile platform to test and integrate key cellular functions outside of living cells. It enables the rapid prototyping of biological components, such as DNA, RNA, and proteins, in a highly controlled environment, allowing researchers to explore and refine the mechanisms of replication, metabolism and compartmentalization without the complexity of a living organism [21,51,52]. By using CFS, scientists can efficiently study the dynamic behaviours of synthetic components and assess their potential for integration into protocells. Furthermore, CFS allows for the development of artificial compartments and membrane-bound processes that mimic organelle functions, advancing our understanding of how to organize cellular activities within a synthetic system [21,36,50].

The modularity and flexibility of CFS are particularly valuable for bridging the gap between synthetic protocells and natural cells, as they facilitate the testing of alternative biological architectures and the creation of life-like systems that could evolve under novel conditions [12,53]. By optimizing protein synthesis, energy generation and compartmentalization in cell-free platforms, researchers are steadily progressing towards creating more sophisticated synthetic cells. Ultimately, CFS provides a powerful tool to not only dissect and re-engineer key features of natural life but also to explore the potential for designing alternative life forms with unique capabilities, bringing us closer to the realization of fully functional synthetic cells [54–58].

**Figure 2.**
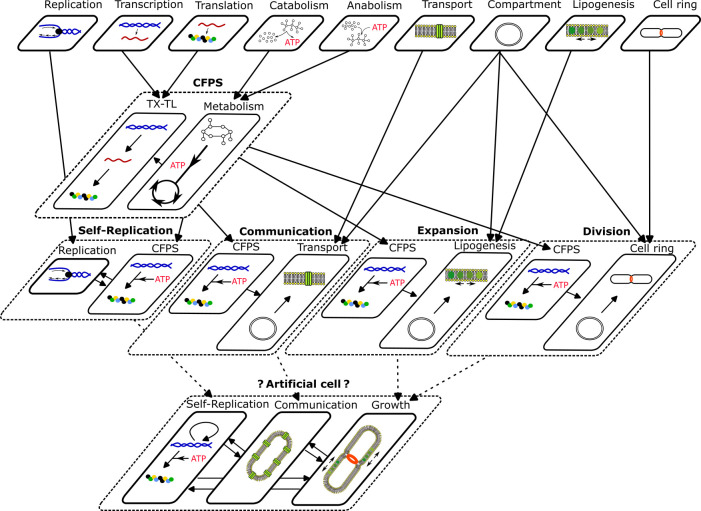
Core modules and hierarchical complexity in artificial cell assembly. From the top to the bottom layers, the modules become increasingly complex, with the initial merging of modules indicated by black arrows. *On the top* of the figure are a selection of fundamental functions divided into nine modules: DNA replication (in blue is DNA, the black dot is the DNA polymerase); transcription (in blue is DNA, in red mRNA); translation (in red is mRNA and the coloured dots represent a protein); catabolism (schematized by a molecule degraded into energy); anabolism (schematized by energy used to produce a molecule); transport (the yellow dots stand for lipids and the green structure for a transporter); compartment (the two-layered circle stands for the lipid bilayer); lipogenesis (the yellow dots stand for lipids, the green dots for the lipogenesis machinery, and two arrows to schematize lipid expansion); and cell ring (the red ring represents the cell ring between two dividing cells). *In the second layer*, the cell-free protein synthesis (CFPS) is composed of the transcription and translation processes (from DNA to protein) with the addition of metabolism (schematized by a sugar consumed to produce energy). *In the third layer*, each module consists of the CFPS with an added higher-level function. Self-replication also contains DNA replication, communication requires compartment and transport, expansion is based on compartmentalization and lipogenesis, and division uses compartment and cell ring. *In the fourth layer*, an artificial cell containing highest-level functions is schematized with self-replication (DNA replication, transcription, translation and metabolism), communication, and cell-division based on a compartment showing transport, lipogenesis and a cell ring.

Next, we explore how CFS has been used to test variants of the aforementioned three core functions of life: information replication, metabolism and cellular compartment (figure 2).

## 2. Information transfer in living systems

The core of information transfer in living cells is the central dogma that constitutes (i) replication to copy DNA instructions from one generation of a cell to another, (ii) transcription to generate messenger RNA from DNA, and (iii) translation to generate the functional workhorses (proteins) from messenger RNAs (mRNAs). Naturally evolved cells tightly regulate these processes to ensure that the right molecules are synthesized at the right time. However, orthogonal components of information transfer systems can function with reduced interaction with the natural cellular machinery [59], gaining some degree of independence from the regular transcription and translation processes, thus allowing for specialized, controlled gene expression without interfering with the cell’s native functions. When used in CFS, these orthogonal systems offer several advantages, including flexibility, tunability, and limited regulation [60]. As CFS lack the cellular environment and compartmentalization of living organisms, they provide an open setting for controlled manipulation and observation of biological reactions, such as DNA replication, transcription, and translation. However, these often come at the cost of reduced efficiency of these processes in CFS in comparison to their *in vivo* counterparts (table 1).

**Table 1.** Rates of information transfer for replication, transcription and translation. (The rates of information transfer for each process in natural cells versus cell-free systems are compared side by side.)

process	rate in natural cells	rate in cell-free systems	references
replication	600−611 nt s^−1^		[61,62]
		∼20 nt s^−1^	[63]
		∼50 nt s^−1^	[64]^a^
transcription	42 nt s^−1^		[65]
		10 nt s^−1^	[66]
		8.1−11.5 nt s^−1^	[33]
		12−30 nt s^−1^	[67]^b^
translation	14 aa s^−1^		[65]
	15 aa s^−1^		[68]
	8 aa s^−1^		[69]
		~1 aa s^−1^	[66]
		5.4−6.3 aa s^−1^	[33]
		>4 aa s^−1^	[70]
		1−2 aa s^−1^	[71]

^a^ phi29 DNA polymerase.

^b^ T7 RNA polymerase.

### (a) DNA replication in cell-free systems

In natural cells, DNA replication requires a network of specific signals, like replication origins, along with various enzymes to ensure faithful DNA duplication and transmission to daughter cells. Key enzymes, such as primases, DNA-dependent DNA polymerase, helicase and topoisomerases, orchestrate the replication process, ensuring high fidelity and efficiency. However, these enzymes are not included in PURE CFS, and lysate-based CFS frequently lack certain enzymatic activities essential for complete replication [72]. For *in vitro* DNA amplification, the most common approach is polymerase chain reaction (PCR). However, PCR requires thermal cycling through extreme temperatures, a condition impractical for natural cells or protocells.

As a result, single-subunit DNA polymerases, which require minimal accessory proteins, are typically preferred for DNA replication in CFS [73]. Researchers have employed the bacteriophage phi29 DNA polymerase for rolling-circle replication, which operates isothermally, making it compatible with CFS and *in vitro* settings where temperature stability is desired [73]. This technique enables DNA replication without the constraints imposed by multiprotein complexes or thermal cycling, facilitating stable and continuous DNA amplification suitable for CFS applications. By coupling phi29 DNA replication with intermittent evolution, self-replication was improved by fivefold in liposomes containing PURE CFS [74].

### (b) Xeno nucleic acids as non-canonical hereditary molecules

Synthetic biology has expanded the scope of genetic information storage by developing XNAs as non-canonical hereditary molecules with alternative sugar-backbone compositions. XNAs also introduce a ‘genetic firewall’, distinguishing artificial life forms from natural biological systems and thereby reducing the risk of unintended genetic crossover [75]. They not only serve as reservoirs for genetic information but also possess functional capabilities. For example, certain XNAs can act as ligand aptamers or function as catalysts—such as ribozymes and DNAzymes—enabling a variety of chemical reactions that mimic the biochemical versatility of natural nucleic acids [76]. This functional versatility has positioned XNAs as promising candidates for therapeutic applications [77].

Given their potential for information storage, engineered or evolved polymerases have been developed to replicate XNAs [78]. Modifications introduced into these polymerases often broaden their substrate specificity, allowing replication and maintenance of a wider range of nucleic acid molecules, both natural and synthetic [77,79]. Despite many enzymes being evolved for XNA-templated replication, XNAs still remain very stable molecules owing to the lack of natural nucleases to degrade them, making them especially suitable for synthetic biology and biotechnology applications [80].

### (c) Transcription in cell-free systems

The transition from DNA information storage to functional expression requires transcription, whereby DNA is transcribed into RNA. This is an essential intermediate step in biological information transfer, as it generates the RNA templates required for protein synthesis. To analyse transcriptional processes in CFS, researchers have tested various CFS lysates and hybrid systems using promoter libraries, allowing for comparative studies of transcription efficiency and specificity [81]. Promoter libraries from viral sources, such as the T7 promoter, have been evaluated in both *in vitro* and CFS settings, providing insights into promoter activity across different system types [82–84].

Buffer composition in CFS has been shown to affect transcriptional efficiency, and buffer optimization studies provide insights into these effects, particularly when comparing transcription from circular versus linear DNA templates [85]. Such findings are instrumental for the rapid prototyping of biosensors in CFS, especially when engineered transcription factors are used to enhance system sensitivity and specificity [86].

### (d) Translation and engineering of translation machinery

Advancements in cellular and CFS technology have significantly expanded the capabilities of translation machinery, facilitating the exploration of translational processes beyond the limitations of natural systems. By engineering translation systems to use quadruplet codons, translation can now incorporate an expanded set of amino acids, thereby increasing the diversity of synthesized proteins [75]. In some cases, co-expression in CFS has led to the formation of novel protein complexes, such as a nano-doughnut structure formed by co-expressing two distinct proteins [87]. Additionally, CFS have been instrumental in the rapid screening and structural analysis of self-assembling virus-like particles (VLPs), such as VLPs containing the Hepatitis B core protein (HBc), and for the expression of complex membrane proteins [88–90].

CFS platforms have also been used to introduce chemical modifications to proteins that are challenging to achieve in living cells. Examples include glycosylation and bio-orthogonal tagging [58,91,92], as well as phosphorylation [93]. CFS systems facilitate the expression of antimicrobial peptides [94] and ribosomally synthesized and post-translationally modified peptides (RiPPs) [95]. Engineered orthogonal ribosomes improve translation efficiency within CFS, allowing for increased protein yield and enabling protein expression independent of cellular viability constraints [43]. After achieving minimal activity, these ribosomes can be subjected to evolutionary rounds of modification and selection for further optimization [96].

Using an unorthodox approach to artificial cells, Karzbrun *et al.* [97] developed a highly miniaturized CFS system on a silicon chip. This system successfully recreated oscillating protein expression patterns and gradients, marking a significant step towards a different kind of ‘artificial cells’ on a chip. Furthermore, Kosaka *et al.* [98] have recently reported a significant advance in ribosome biogenesis, where they successfully achieved *in vitro* expression of both small and large ribosomal subunits in CFS. Using a sensitive reporter assay, they demonstrated autonomous transcription, translation, processing and assembly of ribosomes in a single reaction, furthering the fundamental understanding of self-replication and the creation of artificial cells.

Building on these advances, researchers are further expanding the potential of CFS by engineering transfer RNA (tRNA) molecules that enable incorporation of non-canonical amino acids, including cyclic amino acids, into synthesized proteins, which expands the functional diversity of CFS-generated proteins [99]. Engineered tRNAs can also substitute specific amino acids with surrogates, adding another layer of genetic separation from natural systems [100]. Many of these modifications expand the diversity of proteins produced for industrial applications, but it is unclear how they would fit into an alternative form of life.

### (e) Alternative information transfer: the mirror-image central dogma

Mirror life includes all the molecules and machinery necessary to synthesize and assemble l-nucleic acids and d-amino acids, instead of their natural counterparts the d-nucleic acids and l-amino acids. Xu & Zhu [101] recently developed a mirror-image T7 RNA polymerase to produce L-RNAs as well as mirror versions of 5S, 16S and 23S ribosomal RNAs, marking an important step towards realizing the mirror-image central dogma, and the next step would be the construction of a mirror-image of the full translation process to produce d-proteins. Synthesizing mirror ribosomal proteins, tRNAs, aminoacyl tRNA synthetases and translation factors presents a substantial challenge. CFS could be particularly valuable for synthesizing, assembling and optimizing these components, and for integrating them with mirror DNA polymerase for replication and mirror T7 RNA polymerase for transcription [102]. As suggested by Rohden *et al.* [103] a fully integrated system for mirror replication, transcription and translation to explore a substantially alternative form of life will very likely be first implemented in CFS owing to the essentiality constraint of these processes in a living cell.

## 3. Metabolism in cell-free systems

Metabolism is the function of the cell that combines all reactions involved in the conversion of nutrients into energy, to run cellular processes, to assemble cellular building blocks (proteins, lipids, carbohydrates and nucleic acids), and to remove waste. It is usually classified into catabolism, the breaking down of compounds, and anabolism, the synthesis of compounds from smaller building blocks. Several core metabolic pathways, such as the citric acid cycle, are very similar across different species, which is thought to indicate their importance and their emergence early in the evolutionary process [104,105].

### (a) A minimal metabolism

A cell’s metabolism requires several components taken in from the environment, such as substrates like carbon, nitrogen, phosphorus and sulphur for the biosynthesis of proteins, lipids, nucleic acids and amino acids [104–107]. Another crucial component is ATP which acts as an energy carrier to fuel reactions for substrate conversion, transport or cellular maintenance. Reducing equivalents like NADH and NADPH are required for reducing carbon and nitrogen, and cofactors and coenzymes, such as NAD+, NADP+ and coenzyme A, are essential for electron transfer in redox reactions as well as the synthesis of molecules like fatty acids [106]. The metabolic networks of naturally evolved cells have large redundancies, with several interconnected pathways leading to the same products, facilitating robustness and adaptability [107]. Consequently, many of these pathways can be deleted without affecting the cell’s viability.

What could the metabolic network of a minimal cell look like after all the non-essential reactions have been removed? This question has been addressed through a series of systems biology approaches, complemented by improvements in genomic technologies [108]. Gil *et al.* [109] identified a minimal metabolic network consisting of 50 enzymatic reactions. In another study, the authors performed data mining to identify a minimal anabolic network and the metabolome for a reductive chemoautotroph which consisted of 287 metabolites [110]. Studying the reduced-genome bacterium *Mycoplasma pneumoniae* led to the identification of a minimal metabolic network of 189 reactions catalysed by 129 enzymes [111]. However, a lysate-based CFS from the reduced-genome *Mycoplasma mycoides* showed only modest gene expression [112], possibly because a reduced metabolic network reduces the system’s robustness.

Apart from the components necessary for substrate conversion and energy transfer, another essential part of metabolism is the removal of waste, such as the CO_2_ formed during cellular respiration and biosynthesis, ammonia or urea, excess protons that accumulate during glycolysis or the electron transport chain, as well as heat released during metabolic reactions [113]. A synthetic cell containing a metabolic component should be able to combine these substrates, energy carriers, and waste removal mechanisms, in order to be self-sustainable. However, if only specific functions are to be sustained for a limited amount of time, as is the case for many protein expression CFSes, a subset of these metabolic reactions may be sufficient [114].

### (b) Balancing energy consumption and regeneration in cell-free systems

One of the main challenges with CFS productivity, which is also relevant when assembling a synthetic cell bottom-up, is the difficulty in maintaining a balance of energy production and consumption. Failure in energy regeneration can prematurely halt the system or lead to the accumulation of potentially toxic byproducts, or both. For example, the accumulation of inorganic phosphate in the reaction leads to a decrease in Mg^2+^ concentration, thus halting all protein synthesis. An excess of NTPs inhibits CFS [115], which can in turn be rescued by Mg^2+^ supplementation [116]. A mismatch in the production and consumption rates has a knock-on effect on CFS efficiency. Rapid availability of glucose in the CFS buffer can result in accumulation of organic acids that reduce pH in the system to bring all reactions to a halt, whereas using glycogen and soluble starch prolong reaction time from 8 to 12 h by slowing down glucose availability [117]. The most efficient energy source for ATP in *E. coli* lysates is 3-phosphoglycerate (3-PGA) that leads to 2.3 mg ml^−1^ of protein synthesis by enzymatic recycling of inorganic phosphate by maltose or maltodextrin catabolism [118]. A later system enabled endogenous phosphate recycling by low cost hexametaphosphate supplement that acts as phosphate donor together with maltodextrin, relying solely on the metabolic enzymes already present in the *E. coli* extract and still reaching 1.65 mg ml^−1^ protein yield [119]. Several such interventions at the level of CFS metabolism have been necessary to improve protein expression yields [120].

### (c) Integrating energy regeneration with metabolism

In all of the above, the sources of energy are supplied as chemical moieties in the ‘energy mix’ added to the CFS. To construct self-sufficient synthetic cells, efforts have focused on integrating energy regeneration with metabolism while maintaining external control to keep the energy regeneration constant. One approach involves light-controlled bacteriorhodopsin production in proteoliposome membranes [121]. Bacteriorhodopsin generates H^+^, which powers ATP synthesis from ADP by an ATP synthase. This ATP fuels transcription and translation, as well as GDP-to-GTP conversion to sustain bacteriorhodopsin production through positive feedback. Additionally, ATP synthesis via creatine phosphate enables inorganic phosphate recycling. However, this system has not yet been integrated within artificial cells, and lacks coupling with nutrient uptake or waste removal.

In 2018, Beneyton *et al.* [122] demonstrated a self-sustained metabolic system in a microcompartment (inverted membrane vesicle (IMV)) by integrating an NAD-dependent enzymatic reaction (glucose-6-phosphate dehydrogenase (G6PDH)) with an NAD-regeneration module. They showed that metabolic activity continues as long as glucose-6-phosphate (G6P) is present but ceases upon substrate depletion; however, it can be re-activated through external addition of G6P. The IMVs in this study are derived from small fragments of *E. coli* membranes that naturally contain ATP synthase and NADH oxidation activities. Inspired by this, Biner *et al.* [11] developed a system in 2020 for constant ATP supply. Their minimal respiratory system used NADH as fuel to generate ATP from ADP and inorganic phosphate, supporting NAD+-dependent upstream metabolism and ATP-powered downstream processes. By co-reconstituting mitochondrial complex I and an F-type ATP synthase into nanosized liposomes, they formed a minimal artificial ‘organelle’ mimicking mitochondrial respiration. This system successfully drove cell-free protein expression using NAD+-linked substrates like ethanol or lactate, though byproduct removal (e.g., CO_2_, ammonia, protons) remains unaddressed.

Jewett *et al.* [123] demonstrated that a CFS-based system could co-activate multiple biochemical networks in a single reaction. Previously constrained by factors such as regulation, reaction conditions, compartmentalization and resource competition, they employed a cytomim system (*E. coli* crude extract CFS) to co-activate central catabolism, oxidative phosphorylation, and protein synthesis. Here, glutamate supplied reducing equivalents (NADH) via the tricarboxylic acid (TCA) cycle, while IMVs converted these into ATP through oxidative phosphorylation, driving transcription and translation. However, the membrane was derived from natural *E. coli* cells rather than synthesized via a bottom-up approach. A possible enhancement could combine this TCA cycle with the previously mentioned ATPase inserted into a synthetic membrane, using an intermediate enzyme to convert NADH into H^+^ for ATP synthesis. The required ion-impermeable membrane could be constructed using PDMS-g-PEO, as proposed in Marušič *et al*. [124].

These examples illustrate how CFS metabolism fuels gene expression and synthetic genetic circuits [125–128]. Additionally, CFS-based metabolic engineering has been used for bioproduction, including 3-hydroxybutyrate synthesis [47] and enzyme optimization for biofuels like n-butanol and 2,3-butanediol biosynthesis, circumventing toxicity issues in living cells [34,129]. By enabling the efficient screening and optimization of metabolic pathways, CFS supports faster development cycles for bio-based production systems. In summary, CFS (particularly lysate-based systems) retain essential cellular biochemistries, such as the TCA cycle [6], which can be adapted for alternative reactions. Beyond synthetic life, basal metabolism can be repurposed for industrial applications, positioning CFS metabolism as a versatile platform for synthetic biology and metabolic engineering.

### (d) An alternative metabolism?

While reconstituting a minimal set of cellular metabolic reactions in CFS is a promising route to synthetic life, hints to an alternative route may also exist in non-enzymatic prebiotic metabolism. Several studies have explored early developments in the context of the ‘metabolism-first’ hypothesis of origin of life [130]. Interestingly, Ralser [131] reported the degradation of several intermediate metabolic products in thermophiles in high-temperature conditions. When testing the impact of typical Archean sediment components (such as salts and metal ions) on glycolytic and pentose phosphate pathway intermediates, a high conversion rate of intermediates into each other was observed when Fe(II) was added to the reaction mixtures. Ralser [131] connects this hypothesis with the idea of a ‘prebiotic soup’, suggesting that a pool of organic molecules as well as salts and metal ions must have reacted with each other before evolving into enzymatic metabolism. Yet, bottom-up chemical approaches have failed to discover new/alternative metabolism-like reactions that are also mutually compatible. This is in part owing to extreme differences in reaction conditions (e.g. temperature and pH) in between steps, rendering the discovered metabolic reactions unfeasible in a biological context. As these reactions are also highly sensitive to changing reaction conditions, evolvability is also dramatically reduced. Perhaps, if a mix of enzymatic and non-enzymatic metabolic reactions is incorporated in CFS, it could facilitate the establishment of the minimal metabolism essential for life.

## 4. Cellular and subcellular compartments

Deducing through detailed phylogenomic analysis to ancient, now extinct life forms, it becomes clear that even LUCA was probably already quite complex, and conceptually not very different from modern lifeforms [132]. In the emergence of life as we know it today, compartmentalization played an important role by creating a contained and dynamically controlled environment for biochemical evolution [133,134]. The compartments were made of amphiphilic molecules that can self-assemble into bilayer structures that help in capturing energy and transporting nutrients. Also, environmental conditions could have helped in membrane stability by providing adequate temperature and salinity favourable for these compartments. Some experimental models can demonstrate these kinds of vesicles that resemble the early cellular functions [135]. For example, Montmorillonite clay can catalyse the formation of fatty acid vesicles, providing mechanics for the formation of compartments in a prebiotic form. These kinds of vesicles could encapsulate catalytically active surfaces, providing an example of how early-life compartments might have formed [136]. By separating the biochemical processes, compartments prevent interference between competing reactions and allow the selective exchange of compounds between the living system and the environment [137]. Probably, LUCA already had a confined compartmentalized system that allowed replication to evolve [138].

In current living systems, compartments enable the separation of metabolic pathways, gene expression and interaction with the environment through different types of membranes, which promotes efficiency and self-sustainability of the living systems. However, are compartments essential for living systems, or perhaps simpler synthetic systems can be functionally sustained without them, albeit with reduced efficiency [139]?

### (a) Coacervates and biological condensates

Coacervates are phase-separated droplets without membranes, hypothesized by Oparin & Gladilin [140] as precursors to the first cells on Earth. They consist of concentrated chemical spaces where catalytic reactions can occur, enabling progressively more complex structures and providing insights into how primitive membrane-free compartments may have evolved before the lipid membrane structure [141,142]. Other protocell hypotheses include lipid or polymer vesicles, polyelectrolyte microcapsules, colloidosomes, proteinosomes and supercritical carbon dioxide [143].

Laboratory studies have explored the role of phase separation in facilitating biochemical reactions for primitive metabolism. In 2018, Nakashima *et al.* [144] developed a system where enzyme-catalysed reactions controlled reversible coacervate formation via ATP and ADP partitioning. Two enzymes in this system, pyruvate kinase and hexokinase, function under conditions where ATP condenses into droplets with a polycation (poly-l-lysine), whereas ADP remains in solution. By varying the ratio of the enzyme substrates (phosphoenolpyruvate and d-glucose) the ratio of the condensed ATP and ADP could be varied, thereby controlling the size of the coacervate. In 2020, Bhattacharya *et al.* [145] designed lipid sponge droplets mimicking organelles, allowing material exchange and protein incorporation. A later study used an autocatalytic reaction to regulate hierarchical formation and behaviour of coacervate droplets [146].

In addition to growth, coacervates have been shown to support transcription and translation. Researchers maintained GFP synthesis under varying buffer and lysate concentrations, affecting coacervate size via changes in molecular crowding [36]. Another study also successfully performed *in vitro* transcription and translation of GFP in biomolecular condensates [147]. Additionally, metabolically active coacervates formed from prebiotic metabolites and oligoarginine enhanced NADH oxidation owing to high local concentrations of NADH and ferricyanide [148]. Intracellular biomolecular condensates share properties with coacervates but occur inside cells because of liquid–liquid phase separation [149–151]. These condensates facilitate biochemical reactions without compartmentalization and aid gene regulation by concentrating enzymes and substrates in specific locations inside the cells. Control methods include substrate diffusion from the environment through a vesicle membrane [152], or the use of artificial shape-shifting proteins [153].

The above examples suggest that key cellular functions—information transfer, metabolism and growth—can occur in localized regions without membranes. However, to our knowledge, no study has combined all these functions into one membrane-less system. A compartment may be necessary to maintain far-from-equilibrium conditions, prevent resource parasitism, and enable heritable information transfer for evolution across generations [154].

### (b) Experimental models of primitive cellular compartments

Alternative ways to achieve compartmentalization, which shapes synthetic cells and spatially separates biochemical reactions inside the cell, have been studied. In most living cells, these compartments are made up of lipid-bound structures [155]. However, compartments used to build synthetic cells may be both *membrane-bound* or *membrane-less*.

Lipid-bound membranes are the most common example of membrane-bound compartments, which exist in the form of vesicles, micelles or liposomes. However, the use of non-phospholipid membranes has also been explored through the incorporation of alternative membrane components, such as block copolymers. These polymers consist of hydrophobic and hydrophilic blocks, which can self-assemble into vesicle structures similar to lipid bilayers. These vesicles called polymersomes exhibit increased stability compared to natural phospholipid membranes and can be engineered to have tunable properties, such as permeability and mechanical strength [156].

Another experimental way to reconstitute and restructure alternative life forms is by using microfluidic droplets consisting of water-in-oil emulsions which resemble liposomes [157]. While these membrane-free vesicles cannot recapitulate the complexity and self-sustainability of the naturally formed living forms, microfluidic droplets can be used to mimic a cell-like environment. They can be designed to have a flow of nutrients and a depletion system for removing byproducts to mimic the biochemical dynamics of living cells [158,159]. Incorporation of a reaction-diffusion buffer in microfluidic droplets enabled the longevity of biochemical reactions in the synthetic cell-like environment [160]. The buffer added was important in maintaining the protein production by the already included genetic circuits. The compartments were controlled in a way that would allow the diffusion of the synthesized protein. By controlling the concentration of the reactants, the conditions of the system were maintained to obtain a steady state to express the protein.

### (c) Compartmentalization and signalling

Creating synthetic cells capable of sensing and responding to environmental stimuli requires the insertion of transporter proteins while maintaining membrane integrity. One approach involves fusing oppositely charged proteoliposomes, each containing one membrane protein (such as ATP synthase, and bo3 oxidase), to form larger liposomes with both proteins [161]. However, vesicle fusion can lead to content leakage. An alternative involves proteins that self-integrate into membranes. In 2004, Noireaux & Libchaber [162] encapsulated an *E. coli* CFS in a phospholipid vesicle, where a transcription-translation system (TX-TL) produced α-haemolysin, a pore-forming protein, enabling nutrient influx, oxygen diffusion and osmotic pressure release, prolonging protein production. Selective permeability was demonstrated using fluorescent small molecules, but it was not actively controlled like it is in natural cells. Such pore formation, while useful, also risks excessive permeability and membrane instability. Membrane semipermeability is critical for regulating nutrient import, waste export and ion concentration, essential for metabolism, growth and homoeostasis. However, maintaining controlled permeability without regulatory mechanisms remains a challenge.

Marušič *et al*. [124] successfully integrated cytochrome *bo3* ubiquinol oxidase into a synthetic PDMS-based membrane. This protein oxidizes ubiquinone, reduces oxygen and pumps protons. While proton pumping was observed inside giant unilamellar vesicles (GUVs), the full respiratory chain on a synthetic membrane remains to be achieved. Protein reconstitution in polymer membranes faces limitations owing to thickness, viscosity, rigidity and hydrophobicity, which hinder proper protein orientation and proton gradient formation [163]. Overall, synthetic membrane design must balance permeability, stability and functional protein integration to replicate key biological processes.

## 5. Module combinations: opportunities and pitfalls

**Figure 3.**
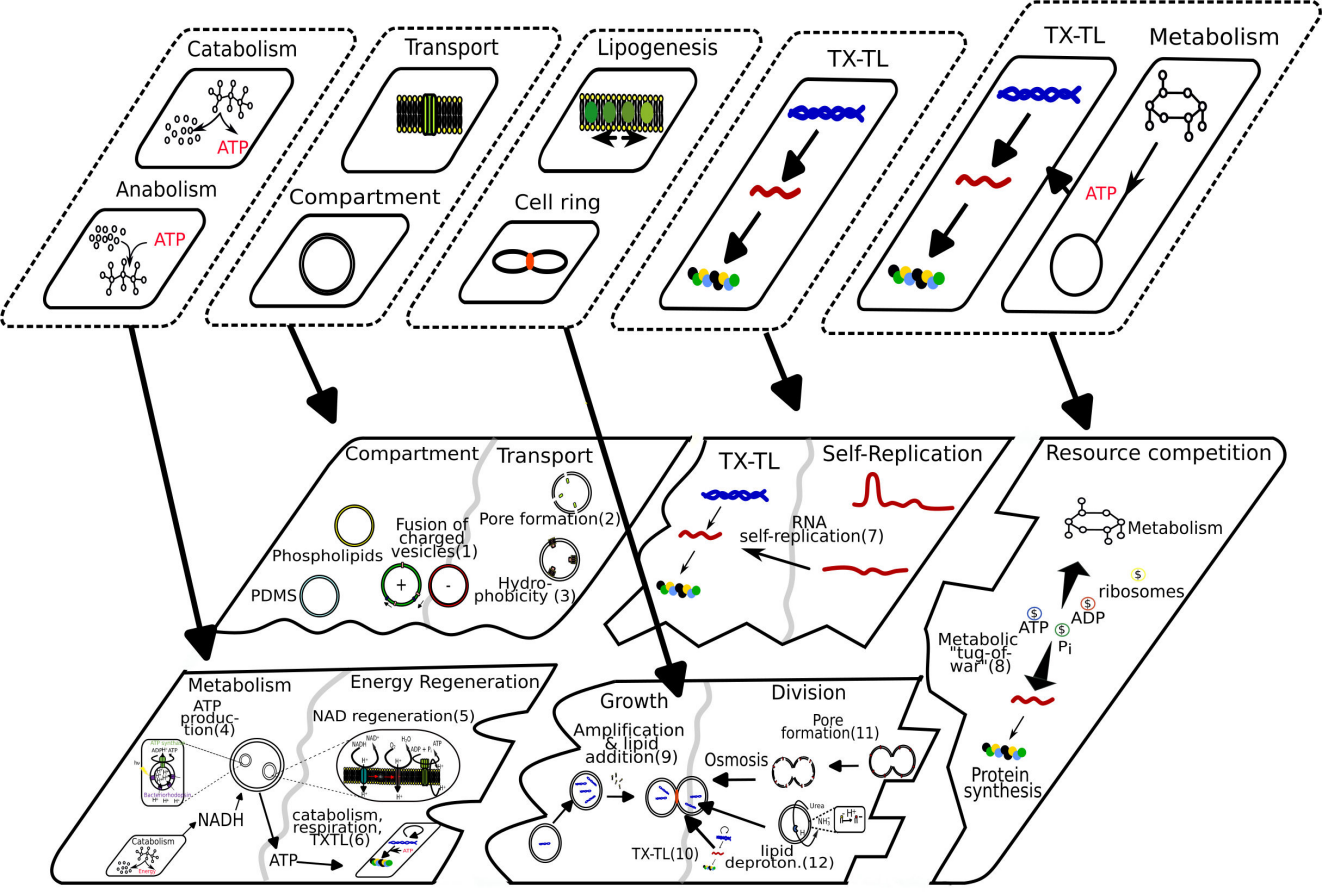
Core modules integrated into an artificial cell. Previously combined modules (see figure 2) are assembled to form an artificial cell. Below, functional module pairs that have been successfully combined are shown as fused tiles, separated by grey lines. Successful combined modules are: compartmentalization and transport; TX-TL and self-replication; metabolism and energy regeneration; and growth and division. Resource competition between all cellular processes is shown as a separate tile on the right. Examples of these successful combination attempts are depicted as numbers in parentheses, which correspond to the following references: 1: [161]; 2: [162]; 3: [164]; 4: [121]; 5: [122]; 6: [123]; 7: [165,166]; 8: [167]; 9: [168]; 10: [53]; 11: [169]; 12: [170]. Functional incompatibilities and constraints when attempting to connect multiple tiles together are depicted as incompatible edge borders and gaps between tiles.

As seen before, a set of core functions (information replication, metabolism and compartments) are essential for life [4,5]. When building synthetic cells bottom-up, these functions need to be combined together to function in a coherent system. However, many combinations of spaces have been shown to remain inaccessible, either owing to functional incompatibilities, such as pH, temperature, spatial or phase separation, or owing to a competition for resources (figure 3). In the following section, we will elaborate on these constraints with some examples.

### (a) Growth and division

Growth and division, intrinsically connected in nature, are fundamental to cellular life and numerous efforts have been made to integrate both into synthetic cells for autonomous reproduction. A well-known example combines self-reproducing GUVs with DNA amplification via PCR, requiring only DNA polymerase and heat-resistant GUVs [168]. In order to create ‘self-reproducing’ vesicles, vesicular membrane precursors were added after amplification, distributing DNA to daughter vesicles. They also observed that amplifying the DNA accelerated division. However, repeated thermal cycles can alter membrane composition. Another approach used phospholipid-oleic acid GUVs, where enzymatic hydrolysis of urea triggered internal pH changes, altering oleic acid protonation. This created electrostatic repulsion causing surface expansion of the inner layer, which together with osmotic stress ultimately led to division [170]. However, the daughter vesicles remained connected, requiring a laser to complete the separation. Furthermore, division occurred only once and was often asymmetric.

In 2021, Dreher *et al.* [169] used GUVs producing the self-assembling chlorin e6 (Ce6) protein. When exposed to light, Ce6-induced peroxidation created membrane pores, facilitating cargo release and division. Another strategy involved a synthetic membrane of triazole phospholipids with self-reproducing catalysts (oligotriazole ligands, specifically tris-(lauryl triazole) amine) that expanded membranes via copper-catalysed reactions [171], and led to the budding off of small daughter vesicles. The *E. coli* divisome consists of the MinCDE protein system which guides the assembly and positioning of a contractile FtsZ ring and its membrane adaptor FtsA. When expressed in lipid vesicles with PURE CFS, these proteins induced ring formation and vesicle shape was transformed [53], though division did not proceed autonomously, possibly owing to the absence of mechanical cell wall expansion. In this regard, this minimal system is similar to DNA-free minicells produced by asymmetric cell division in a number of bacterial species [172,173]. Integration of lipid synthesis machinery into synthetic membranes has also been attempted, allowing precursor condensation and insertion, causing vesicle perturbation, division or internal vesicle release [174,175].

Despite these advances, sustained growth and division of the synthetic cell remain challenging owing to membrane composition changes, uncontrolled destabilization and the need for external intervention. Further refinements are needed to achieve fully autonomous synthetic cell division.

### (b) Resource competition

Resources, such as ADP, ATP, Pi, ribosomes and RNA polymerase, are required to power steps affecting all major processes in the cell, such as gene expression, metabolism, growth and replication. Here, we elaborate specifically on the resource competition between transcription and translation. It has been shown that transcriptional and translational processes compete for ATP [176]. Using a lysate-based CFS, the authors studied energy molecules powering TX-TL and fuel molecules that regenerate energy through the central metabolism. They found a compensatory interaction between the TX-TL components Mg^2+^ and 3-phosphoglycerate (3-PGA, which is used to regenerate ATP). If one component’s concentration is increased, the others must be increased simultaneously as well to maintain optimal translation.

Furthermore, maximum mRNA and protein production occurred in conditions of Mg^2+^ and 3-PGA that were contrary to the expectation, suggesting a coupling of TX and TL. Under translation inhibition, transcriptional output was constant across all Mg^2+^ and 3-PGA concentrations. In a translation-only system, maximum protein production occurred in the previously found optimal regime of Mg^2+^ and 3-PGA. The location and slope of the trade-off curve were determined strongly by DNA concentration, cell lysate batch and the fraction of cell lysate in a reaction. Finally, in systems where additional energy is supplied and where a fuel source is absent, the trade-off is absent. This indicates that the TX-TL dynamics are strongly dependent on Mg^2+^.

Similarly, resource competition between glycolysis and translation has been observed [167]. For a long time, competition was not thought to be a problem in codependent biological systems, e.g. glycolysis (energy-supplying) and translation (energy-consuming). In this work, the authors reconstituted both processes using purified proteins in a CFS. They observed a ‘metabolic tug-of-war’ between glycolysis and translation based on their reaction rates, with successful coupling being dependent on the initial substrate concentration. They use an ATPase to degrade ATP, which promotes strong glycolysis, which in turn produces ATP to fuel translation. Showing that this approach works, even though ATPase normally inhibits translation, underlines the coupling effect between glycolysis and translation.

With the goal of creating synthetic cells, work has been done to combine synthetic ribosomes with synthetic RNA polymerase [177]. These synthetic processes interfered with each other due to resource competition for RNA substrates or cofactors required for transcription, resulting in reduced efficiency or complete inhibition of the transcription process.

### (c) Incompatibility between natural and non-natural components

Incompatibilities can arise not only when combining natural components in a synthetic cell, but also when interfacing natural and non-natural components. One form of incompatibility comes from placing natural components in a non-natural context, such as *E. coli* gene expression machinery (RNA polymerase, ribosomes) in a non-natural CFS buffer (figure 1). Being outside of their naturally optimized *in vivo* context, these components often have reduced efficiency (table 1).

A different form of incompatibility arises when combining natural and non-natural components in a new context. An example of this is the attempt to build protocells with natural lipid-based membranes and evolved/designed RNA for replication [178]. Here, it was shown that often reaction conditions are incompatible and various efforts had to be made to overcome these challenges. Whereas non-enzymatic RNA polymerization and ribozyme activity require high concentrations of divalent cations for stability [179], fatty acid membranes are unstable at those concentrations [180]. Solutions for these incompatibilities have been explored, such as careful control of the ion concentration in the environment, to find a balance between the needs of RNA replication with the stability of the vesicle. Another interesting approach used in these natural–synthetic hybrid protocells involves coupling both components in order to make them dependent upon each other and move towards a protocell capable of evolution [178]. Protocells can achieve this through processes like osmotic pressure, or by linking genome replication to membrane growth via ribozyme activity. The challenge lies in balancing genome replication and compartment replication to avoid overproduction of empty cells.

All in all, interactions between natural components outside of their usual context or natural and non-natural components are still poorly understood and unpredictable. However, with a combination of trial-and-error methodologies and rational balancing of the components, it may be possible to optimize functionality [44,178].

## 6. Discussion

CFS have been shown to have the properties required to enable the construction of a variety of alternative functions, covering all the essential processes necessary to build a living system, from self-replication to cell division (figure 2). Nevertheless, a major issue in reconstructing alternative life remains the lack of compatibility between different approaches used to build artificial modules [181–184]. Most current systems use transcription and translation processes to produce specific synthetic pathways, but these pathways cannot always be connected to new modules. Integrating modules would require extensive redesign to ensure they can recognize and cooperate with one another without depleting shared resources, having time-synchronization issues, or overcrowding. Designing pathways in isolation and attempting to combine them later has shown limited success owing to the context-dependent nature of each pathway’s functionality.

To address these challenges, a few key strategies could improve interoperability across synthetic modules. Potential solutions include standardizing protocols, improving communication between research teams, and creating better interfaces for system integration [185]. The FAIR principles (findable, accessible, interoperable, reusable), generally applied to scientific data management, provide a useful framework for achieving this standardization, fostering data sharing and module compatibility [186,187].

Promising results from projects that prioritize interconnectivity demonstrate that multi-module systems can indeed work together effectively. For example, in a study by Giaveri *et al.* [27], an interconnected approach allowed the development of an artificial metabolism capable of both fixing carbon dioxide and functioning alongside transcription/translation processes. A recent preprint by Sierra *et al.* [188] demonstrates integration of metabolic and genetic networks. The authors engineered phospholipid vesicles that perform transcription-translation of a synthetic genome encoding six proteins, self-replication of this genetic programme and membrane synthesis. Eventually, they successfully integrated these modules and offered to optimize them through directed evolution. These advances illustrate that with careful planning, it is possible to design synthetic biological modules that can integrate and operate collaboratively within a unified system.

Yet, a major issue in constructing synthetic cells is the lack of knowledge about many regulatory mechanisms and gene functions. Even in a minimal synthetic genome [189], there are genes with unknown or partially understood roles, a phenomenon complicated by pleiotropy where a single gene influences multiple traits [190]. Additionally, even attempts to reconstruct natural cells highlight these challenges. For instance, introducing an *E. coli* genome into its own lysate and encapsulating it fails to restart a living organism [191]. This incomplete understanding hinders the ability to fully ‘boot up’ a synthetic cell genome, as the complexity of life goes far beyond merely assembling known parts. Filling these knowledge gaps is crucial for advancing synthetic cell design and functionality.

Another potential solution is to incorporate adaptability and evolution within the synthetic cell design paradigm. By using this approach on modules that are only partially connected through transcription, translation, and resource competition, directed evolution could help resolve incompatibilities, eliminate constraints and identify effective module combinations [192]. Promisingly, the artificial life community has already embraced open-endedness as a key design approach [193], which emphasizes novelty of exploratory designs for biology as being equally important to meeting specific goals [194]. Such novelty exploration may be the solution to the module incompatibilities and interface gaps (figure 3) hitherto seen with the rational approaches towards building the synthetic cell.

## Data Availability

This article has no additional data.
